# A systematic review of patient-reported outcome measures in paediatric endocrinology

**DOI:** 10.1186/s12902-022-01099-z

**Published:** 2022-07-15

**Authors:** Richard G. McGee, Edward Y. B. Zhang, Jennifer J. G. Tan, Aiden C. K. Cheung, Matthew P. Garvey

**Affiliations:** 1 Department of Paediatrics, Gosford Hospital, Holden St, Gosford, NSW 2250 Australia; 2 Central Coast Clinical School, The University of Newcastle, 77a Holden St, Gosford Hospital, Gosford, NSW 2250 Australia

**Keywords:** Endocrinology, Patient reported outcome measures, Pediatrics, Child, Type 1 diabetes mellitus, Hypothyroidism

## Abstract

**Context:**

Patient reported outcome measures (PROMs) are useful tools in paediatric endocrinology to gauge health status in children, especially since they are often unable to clearly communicate it themselves. We aimed to systematically search and appraise all available PROMs relevant to paediatric endocrinology and provide a curated resource for health professionals to utilise.

**Evidence acquisition:**

We identified PROMs in paediatric endocrinology by systematically searching the Cochrane Library, MEDLINE, World Health Organisation International Clinical Trials Registry Platform, and the Cumulative Index to Nursing and Allied Health Literature on May 20, 2022. Additional studies were located through hand searching and content area expert contributions. We assessed the quality of each PROM using the COSMIN risk of bias checklist.

**Evidence synthesis:**

We identified 5003 papers in the initial search. After applying exclusion criteria we included seven PROMs in the review. Six PROMs were specific to Type I Diabetes and one to Hypothyroidism. We gave all studies an overall COSMIN score of ‘inadequate’ due to poorly detailed PROM development.

**Conclusion:**

The scope and quality of PROMs in paediatric endocrinology is limited. Further research and development of PROM tools are required in paediatric endocrinology to allow for improved patient care.

**Supplementary Information:**

The online version contains supplementary material available at 10.1186/s12902-022-01099-z.

## Introduction

Patient reported outcomes (PROs) describe the impact of illness and treatments from the patient’s perspective [1]. They can assess a range of outcomes such as health related quality of life, disease symptoms, behaviours and perceptions of treatment [2]. Patient Reported Outcome Measures (PROMs), are the standardised measures of PROs, and are increasingly being used to assess a patient’s health. They promote patient-centred care, engage patients in their healthcare and help clinicians identify and treat illnesses [3, 4]. In gathering patients’ perspectives in a structured format, clinicians can determine the effect of treatments and services on patients. This helps provide optimal interventions, increases compliance and improves patient quality of life [5].

There are some barriers to the effective uptake of PROMs in clinical practice, including the poor methodological quality of some PROMs, lack of standardisation between tools, and difficulty with selection of PROMs for use [5]. There are also barriers to the implementation of PROMs, including a lack of research on evidence-based PROM implementation strategies and applying techniques from implementation science to PROM implementation [6]. Specifically, within paediatric endocrinology, children may struggle to express their health concerns [7]. It is also common but not optimal for parents and carers to act on behalf of paediatric patients [8]. Whilst the literature around patient-parent concordance is scarce, there is evidence of increased compliance with treatment when patients and parents agree [9].

Online databases of PROMs such as the Patient Reported Outcome Measures Information System (PROMIS) have been developed for generalised illnesses, which has proven beneficial to researchers and clinicians in primary care [10, 11]. However, this database doesn’t capture PROMs for specific subsets of the population, such as children with endocrine conditions. Rather, the PROMs relate to the physical health of broader populations and include domains such as pain, mobility and fatigue.

We aimed to assess PROMs applicable and specific to paediatric endocrinology and to provide a curated resource for health professionals to utilise. This allows clinicians in paediatric endocrinology to identify, evaluate and apply the best PROMS for treatment, management and patient care.

## Methods

This study was reported according to the PRISMA 2020 guidelines [12].

### Protocol registration

The protocol for this study was registered with PROSPERO and available from: https://www.crd.york.ac.uk/prospero/display_record.php?ID=CRD42021251386

#### Literature search

We designed our search strategy to be sensitive by developing a disease filter using the Medical Subject Headings (MeSH) ‘endocrinology’ and ‘endocrine system diseases’. We then combined this with pre-existing and validated search filters for paediatric populations and PROMS [13, 14]. We excluded studies relating to adults or animals from the search. We searched the Cochrane Library, MEDLINE, World Health Organisation International Clinical Trials Registry Platform (WHO ICTRP), and the Cumulative Index to Nursing and Allied Health Literature (CINAHL) databases on May 20, 2022. The complete search strategy can be found in Additional file 1. We located additional studies through hand searching reference lists of relevant studies and content area expert contributions.

#### Study selection

We screened studies for eligibility using Covidence software (Covidence systematic review software, Veritas Health Innovation, Melbourne, Australia. Available at www.covidence.org) and excluded duplicate articles. Each study was independently reviewed by two authors from a panel of four (EZ, JT, AC, MG) for eligibility according to our inclusion and exclusion criteria. We resolved any discrepancies about the eligibility of studies by discussion among a panel of four authors (EZ, JT, AC, MG). We recorded the rationale for excluding articles at each stage.

We included studies where (i) PROMs were developed as either the primary or secondary outcome measured, and where (ii) the study population was paediatric patients (under 18 years of age) with an endocrine disorder including, but not limited to, type I and II diabetes mellitus, growth hormone disorders, thyroid disorders, differences of sexual development and adrenal disorders. We excluded studies, which were (i) not available in English, (ii) did not utilise PROMs, (iii) did not pertain to a paediatric study population or included adults in the study population, or (iv) if the PROM was not specifically related to an endocrine disorder.

Once the final studies were selected, two authors from a panel of four (EZ, JT, AC, MG) independently extracted data and performed quality assessments for each study. The data extracted from included studies were: name of PROM tool, endocrine conditions studied, date and location(s) the study was conducted, the number of participants, age and gender of participants, and where participants were recruited from. Data regarding the PROM tools were also extracted and included: suitable population, number of items, mode of completion and reporting, method of development, languages available and reliability in terms of Cronbach’s alpha.

#### Risk of Bias assessment

We identified the development articles for the included PROMs through reference lists of articles included in our full text review. Development articles were then screened for risk of bias using the COnsensus based Standards for the selection of health Measurement INstruments (COSMIN) Risk of Bias checklist [15]. Two authors from a panel of four (EZ, JT, AC, MG) used this checklist independently to evaluate PROM development, content validity, structural validity, internal consistency, cross-cultural validity, reliability, measurement error, criterion validity, construct validity, and responsiveness for each included article. We resolved any discrepancies about the risk of bias through discussion among a panel of four authors. COSMIN uses a “worst score counts” system in which items are given a score of very good, adequate, doubtful or inadequate and the lowest score for any standard is used [15]. We then gave each article an overall risk of bias score.

## Results

**Fig. 1:**
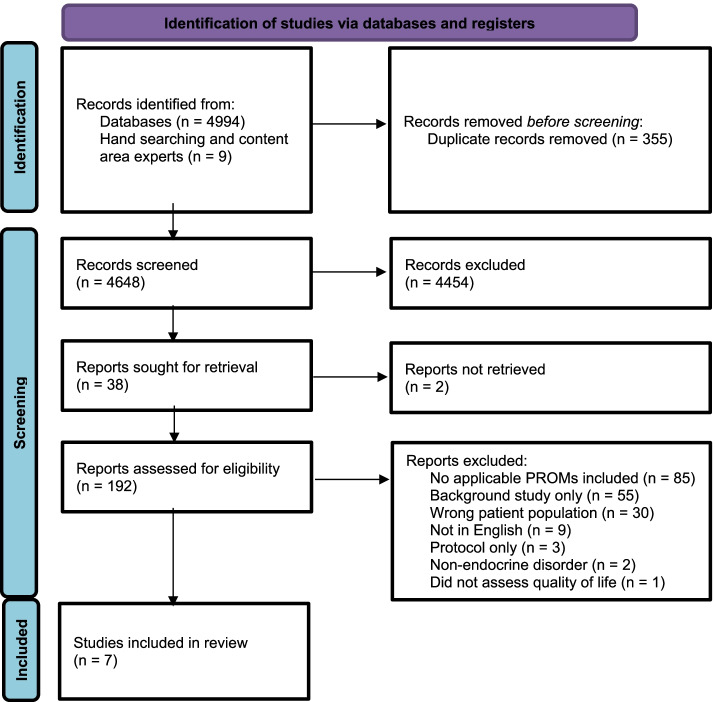
Identification of studies

Our search returned 5003 unique publications, of which 4454 were excluded based on title and abstract screening. A further 185 publications were excluded based on full text review, according to the eligibility criteria. We excluded most of these 185 publications because they did not contain PROMs specifically designed for paediatric endocrinology (*n* = 85). From the full text review, we identified seven studies that met our inclusion criteria, as shown in Fig. 1. These PROM tools were developed for two endocrine conditions; Type I Diabetes Mellitus (TIDM) [16–21] and hypothyroidism [22].

The included studies were conducted between 1998 and 2015 and were mostly conducted in the United States and Europe. The characteristics of the included studies are shown in Table 1. While some studies developed original PROMs [16, 19, 20], three studies modified pre-existing adult PROMs [17, 18, 22]. One PROM combined items from pre-existing paediatric PROMs as well as incorporating original items [21].

**Table 1:** Characteristics of included studies

Patient reported outcome measure	PedsQL Diabetes Module [16]	Problem Areas In Diabetes Scale - Child Version [17]	Diabetes Quality of Life for Youth scale - Short Form [18]	DISABKIDS condition-specific modules [19]	DISABKIDS chronic generic module [20]	Monitoring Individual Needs in Diabetes Youth Questionnaire [21]	Hypothyroidism symptom score [22]
**Endocrine condition(s)**	Type I Diabetes	Type I Diabetes	Type I Diabetes	Asthma, arthritis, epilepsy, cerebral palsy, type I diabetes, atopic dermatitis, cystic fibrosis	Asthma, arthritis, epilepsy, cerebral palsy, type I diabetes, atopic dermatitis, cystic fibrosis	Type I Diabetes	Subclinical Hypothyroidism
**Date conducted**	March 2003	2014–2015	March–August 1998	April–July 2003	2002–2003	2012	December 2014
**Location**	USA	USA	18 countries in Europe, Japan and North America	Austria, France, Germany, Greece, Sweden, Netherlands, United Kingdom	Austria, France, Germany, Greece, Sweden, Netherlands, United Kingdom	Netherlands	Turkey
**Number of participants**	300 children 308 parents	804 children 968 parents	2077 children	Total = 1152 children Diabetes module = 207 children Number of parents not stated	1153 children 1061 parents	84 children online 22 children face to face	27 children
**Age**	Mean = 12.47 +/− 4.04 Range = 2–18 (ages 2–4 were parent responses only)	Mean = 10.3 +/− 1.1 years Range = 8–11.9 years	Mean = 14.3 +/− 2.1 years Range = 10–18 years	Mean = 12.2 +/− 2.8 years Range = 8–16 years	Mean = 12.2 +/− 2.8 years Range = 8–16 years	Mean = 14.4 +/− 2.2 years Range = 10–18 years	Mean = 10 +/− 6.9 years
**Gender**	43.9% Male 56.1% Female	48.4% Male 51.6% Female	52% Male 48% Female	52% Male 48% Female	51% Male 49% Female	48% Male 52% Female	59% Male 41% Female
**Recruitment locations**	Children presenting at hospital based diabetes clinics and children presenting at paediatricians offices for scheduled well-child checks	42 Diabetes ‘camps’ across USA	22 paediatric diabetes centres	Paediatric clinical settings from the 7 participating countries	Paediatric clinical settings from the 7 participating countries	Two paediatric outpatient clinics	Outpatient paediatric endocrinology clinic

We outline the results of the COSMIN Risk of Bias assessment in Table 2 as assessed from the PROM development articles [16–22]. We gave all studies an overall COSMIN score of ‘inadequate’. Measurement error, criterion validity and responsiveness were not reported in any of the studies. Structural validity, internal consistency, and hypothesis testing were considered ‘very good’ for most of the studies, while the other domains were doubtful or inadequate. Reliability was only reported in one study [21]. None of the studies used trained interviewers, or it was doubtful that they did, when presenting questions to subjects. Only three studies explicitly asked patients about at least two of the following factors: relevance, comprehensiveness, and comprehensibility of the PROMs [16, 19, 20]. Only three studies included professional input regarding relevance and comprehensiveness [17, 18, 21]. Moreover, none of the studies clearly described asking both patients and professionals about these domains. Although all papers were deemed inadequate overall, the papers with the best methodological quality, according to the COSMIN checklist, were the short form of the Diabetes Quality of Life for Youth scale [18] and Problem Areas In Diabetes Scale for children [17]. Each of these papers received a score of ‘very good’ in the assessable domains of structural validity, internal consistency, cross-cultural validity, and hypothesis testing for construct validity. The hypothyroidism symptom score was adapted from an adult version of the tool with details on PROM development, which may not accurately reflect the needs of this population [23]. Given the risk of bias of the included studies and the fact that no PROM was validated more than once, the overall certainty of evidence was judged as low.

**Table 2:** COSMIN risk of bias assessment

Patient reported outcome measure	PedsQL Diabetes Module [16]	Problem Areas In Diabetes Scale - Child Version [17]	Diabetes Quality of Life for Youth Scale - Short Form [18]	DISABKIDS Diabetes Specific Modules [19]	DISABKIDS Chronic Generic Module [20]	Monitoring Individual Needs in Diabetes Youth Questionnaire [21]	Hypothyroidism Symptom Score [22]
**Overall COSMIN Score**	Inadequate	Inadequate	Inadequate	Inadequate	Inadequate	Inadequate	Inadequate
**PROM development**	Inadequate	Inadequate	Inadequate	Inadequate	Inadequate	Inadequate	Inadequate
**Content validity**	Doubtful	Doubtful	Doubtful	Doubtful	Doubtful	Doubtful	–
**Structural validity**	Very good	Very good	Very good	Very good	Very good	Inadequate	–
**Internal consistency**	Very good	Very good	Very good	Very good	Very good	Very good	–
**Cross cultural validity / Measurement invariance**	Doubtful	Very good	Very good	–	Adequate	Inadequate	–
**Reliability**	–	–	–	–	–	Adequate	–
**Measurement error**	–	–	–	–	–	–	–
**Criterion Validity**	–	–	–	–	–	–	–
**Hypotheses testing for construct validity**	Very good	Very good	Very good	–	Very good	Doubtful	–
**Responsiveness**	–	–	–	–	–	–	–

‘-’ not assessed based on COSMIN recommendations

We describe the characteristics of the included PROMs in Table 3. Both patient and parent reported outcomes were measured in four instances [16, 17, 19, 20], whilst three studies used only patient reported outcomes [18, 21, 22]. Four PROMs asked patients about quality of life only [17, 19–21], one asked about symptoms only [22], and two asked about both [16, 18]. PedsQL had the youngest child self-reported PROM, with children from the age of 5 years included, however the ⍺-coefficient for internal consistency was only considered reliable in one of five subscales [16]. Six PROMS were flexible in their delivery, providing participants the option to complete the assessments at home [16–21]. Five PROMs were also available in a language other than English [16, 18–21]. The Diabetes Quality of Life for Youth short form was available in fourteen languages [18]. The domains that were well addressed during PROM development included structural validity and internal consistency, with all studies receiving a score of very good in these domains, except for one study in which we could not assess these domains [22], and one that received an inadequate for structural validity [21]. Four studies [17, 19–21] calculated Cronbach’s alpha to be 0.7 or higher, as shown in Table 3, demonstrating satisfactory internal consistency, or interrelatedness between items measured. The other studies [16, 18, 22] either did not calculate it, or had scores below 0.7 in some subscales.

**Table 3:** Characteristics of PROMs

Patient reported outcome measure	PedsQL Diabetes Module [16]	Problem Areas In Diabetes Scale - Child Version [17]	Diabetes Quality of Life for Youth scale - Short Form [18]	DISABKIDS condition- specific modules [19]	DISABKIDS chronic generic module [20]	Monitoring Individual Needs in Diabetes Youth Questionnaire [21]	Hypothyroidism symptom score [22]
**Suitable population**	Patients aged 5–18 years with type I diabetes	Patients aged 8–12 years with type I diabetes	Patients aged 10–18 years with type I diabetes	Patients aged 8–16 years with asthma, arthritis, epilepsy, cerebral palsy, type I diabetes, atopic dermatitis, or cystic fibrosis	Patients aged 8–16 years with asthma, arthritis, epilepsy, cerebral palsy, type I diabetes, atopic dermatitis, or cystic fibrosis	Patients aged 10–18 years with type I diabetes	Children with subclinical hypothyroidism
**Number of items**	28	11 Child self-reported 16 Parent reported	21	Diabetes module = 15	37	36	16
**Mode of completion**	Clinical assessment: Age 5–7: administered by a research assistant Age 8–18: Self-administered Telephone assessment: Questionnaires were read to the child or parent verbatim	Online questionnaire completion	All questionnaires were completed confidentially and returned in a sealed envelope during a routine clinic visit	Completed at the hospital or at home	The questionnaires were filled in by the children and their parents either at hospital or at home and returned by post	Either online questionnaires or semi-structured interviews	Mode of completion not stated
**Method of reporting**	Patient and parent reported	Patient and parent reported	Patient reported only	Patient and parent reported	Patient and parent reported	Patient reported only	Patient reported only
**Method of development**	Focus groups, individual focus interviews, cognitive interviewing, pretesting and field testing	Simplification of language in the teen version of the Problem Areas In Diabetes survey by paediatric psychologists. Field testing and factor analysis performed to remove items for low communalities.	Exploratory and confirmatory factor analysis of the original 52 item DQOLY questionnaire	Literature review, focus groups, cognitive interview, item selection, translations, pilot study, field study, implementation study	Literature review, focus groups, translation, pilot study, field study	Modified from DQOLY-SF, with addition of components from PedsQL, Diabetes Family Conflict Scale (DFCS), Confidence in Diabetes Self-care-Youth (CIDS-Youth) and Eating Disorders Examination- Questionnaire (EDE-Q)	Modified from a hypothyroidism symptom score for adults
**Item categories**	Child self-report: Diabetes symptoms Treatment barriers Treatment adherence Worry Communication Parent proxy-report: Same domains	Child self-report: Emotional burden Regimen specific distress Parent proxy-report: Negative emotions Keeping up with chronic demands Personal regimen-specific distress Child regimen-specific distress	Impact of diabetes Worries about diabetes Satisfaction with treatment Satisfaction with life Health perception	Impact Treatment	Mental independence Mental emotion Social exclusion Social inclusion Physical limitation Physical treatment	Social impact Parents Diabetes control perceptions Responsibility Worries Treatment satisfaction Body image and eating behaviour	Hypothyroidism symptoms
**Languages available**	English and Spanish	English	14 languages (not specified)	German, French, English, Swedish, Greek, Dutch	German, French, English, Swedish, Greek, Dutch	English and Dutch	English
**Reliability as measured by Cronbach’s alpha**	**Child self report** Diabetes symptoms = 0.81 Treatment barriers = 0.66 Treatment adherence = 0.66 Worry = 0.63 Communication = 0.77 **Parent reported** Diabetes symptoms = 0.81 Treatment barriers = 0.68 Treatment adherence = 0.73 Worry = 0.81 Communication = 0.84	**Child self-reported** Overall = 0.91 Emotional burden = 0.86 Regimen-specific distress = 0.87 **Parent reported** Overall = 0.92 Emotional burden = 0.91 Child regimen-related distress = 0.85	Future worries scale = 0.82 Parental influence scale = 0.79 Impact on activities scale = 0.65 Symptom impact scale = 0.51 Impact of treatment scale = 0.47	Overall: 0.75–0.89 Diabetes module: Impact domain = 0.84 Food domain = 0.76 Injections domain = 0.82	0.70–0.87	Overall: 0.8 Social impact = 0.68 Parents = 0.64 Diabetes control perceptions = 0.76 Responsibility = 0.36 Worries = 0.64 Treatment satisfaction = 0.79 Body image and eating behaviour = 0.38 WHO-5 = 0.78	Cronbach’s alpha not calculated

## Discussion

Our systematic review identified seven published PROMs in paediatric endocrinology. As such, the current literature only includes a subset of conditions dealt with by paediatric endocrinologists, i.e. TIDM and hypothyroidism. While the overall methodological quality of the studies was variable, many domains were inadequate and some PROM measures were only adapted from an adult population [17, 18, 22]. PROMs identified varied in their assessment of quality of life, disease related symptoms or both. Although assessment of quality of life may reflect a patient’s disease status and presence of symptoms, it may be more beneficial for PROMs directed at children to have a larger focus on assessing symptoms.

There are few PROMs available in paediatric endocrinology. Other paediatric disciplines face similar challenges. For example, a 2018 systematic review in otolaryngology identified eight PROMs, with only three specific to paediatrics [23]. A 2021 review of PROMs relevant to paediatric orthopaedics found seven PROMs [24]. In comparison, reviews of PROMs designed for adult use in gastroenterology and psychiatry identified 126 and 103 PROMs respectively [25, 26]. A scoping review in 2021 by Churruca et al., found 315 generic and condition specific PROMs across 17 disease groups, with 13 in adult endocrinology [27]. The scarcity of PROMs is not an issue isolated to paediatric endocrinology, but clearly fewer studies relevant to children are published.

The ‘inadequate’ quality rating for all PROMs overall was mainly because of shortfalls related to the domain of PROM development. We largely attribute this to the fact that the COSMIN Risk of Bias checklist was published in 2018, which is after most of these PROMs were developed [15]. These studies do not necessarily report on aspects of the development process as outlined by COSMIN. Similar to our findings, reviews of PROMs in other paediatric fields have recognised that the level of detail provided in PROM development is often insufficient [24, 28–30]. Since we cannot be confident whether these tools can be accurately and reliably used to gauge a child’s health status, it is difficult to recommend any existing PROM for use. The relatively old age of the PROMs reviewed, apart from three [17, 21, 22], indicates a need for PROMs in Paediatric endocrinology to be updated to align with the most recent guidelines for PROM development. Clinicians and researchers should take this into consideration when applying these PROMs to their own practice.

To our knowledge, we are the first group to perform a systematic review of PROMs in paediatric endocrinology. A previous study evaluated health related quality of life questionnaires for adolescents with diabetes and assessed their psychometric properties [31]. This study included both generic and diabetes measures and did not use the COSMIN criteria to evaluate the tools they identified. Our collation of published PROMs relevant to paediatric endocrinology provides details on the key characteristics, strengths and weaknesses of each instrument. Our systematic search and standardised approach to evaluating the quality of each tool enables a comparison between instruments and should assist clinicians when deciding which PROM to use. However, our study was limited by the inherent subjectivity of the COSMIN tool. Another limitation of using the COSMIN tool is that older PROMs were not developed with the COSMIN criteria in mind, which may cause poorer reporting on aspects of the development process. While COSMIN recommends using the GRADE tool to analyse the certainty of evidence, this was not relevant to our study as no PROM was validated more than once and all the studies were deemed to be at risk of bias.

Given the methodological quality of existing PROMs, we should make efforts to develop and validate high-quality PROMs, considering the relevant aspects of the COSMIN risk of bias checklist. Currently, most PROMs are designed for TIDM. There remains a wide scope for researchers to develop PROMs for additional paediatric endocrinological conditions.

## Conclusion

In summary, the value of PROMs in paediatric endocrinology has been under recognised. This review provides a useful resource for health care professionals, but more PROMs need to be developed before we can use them across the spectrum of paediatric endocrinology care.

## Supplementary Information

**Additional file 1.** Search terms.

## Data Availability

All data generated or analysed during this study are included in this published article [and its supplementary information files].

## Abbreviations

PROs: Patient Reported Outcomes
PROMs: Patient Reported Outcome Measures
PROMIS: Patient Reported Outcome Measures Information System
COSMIN: COnsensus-based Standards for the selection of health Measurement INstruments
MeSH: Medical Subject Headings
CINAHL: Cumulative Index to Nursing and Allied Health Literature
WHO-ICTRP: World Health Organisation - International Clinical Trials Registry Platform
PedsQL: Pediatric Quality of Life Inventory
TIDM: Type I Diabetes Mellitus
