# Supplementary material for: A systematic review of patient-reported outcome measures in paediatric endocrinology
Source: BMC Endocr Disord. 2022 Jul 15;22:177. doi: 10.1186/s12902-022-01099-z (PMC9284786; doi:10.1186/s12902-022-01099-z)
Supplement: Supplementary file 1 — Additional file 1. Search terms. [file 12902_2022_1099_MOESM1_ESM.docx]

**Additional file 1. Search terms**

| Databases | MEDLINE, Cochrane library, CINAHL Complete, WHO ICTRP |
| --- | --- |
| 1. Paediatric Population Filter[(12)](https://www.zotero.org/google-docs/?KWEmpC) | Infant/ or Infant*.mp. or infancy.mp. or Newborn*.mp. or Baby*.mp. or Babies.mp. or Neonat*.mp. or Preterm*.mp. or Prematur*.mp. or Postmatur*.mp. or Child/ or Child*.mp. or Schoolchild*.mp. or School age*.mp. or Preschool*.mp. or Kid.mp. or kids.mp. or Toddler*.mp. or Adolescent/ or Adoles*.mp. or Teen*.mp. or Boy*.mp. or Girl*.mp. or Minors/ or Minors*.mp. or Puberty/ or Pubert*.mp. or Pubescen*.mp. or Prepubescen*.mp. or Pediatrics/ or Paediatric*.mp. or Paediatric*.mp. or Peadiatric*.mp. or Schools/ or Nursery school*.mp. or Kindergar*.mp. or Primary school*.mp. or Secondary school*.mp. or Elementary school*.mp. or High school*.mp. or Highschool*.mp. |
| 2. Disease Search Strategy | Endocrinology/ or exp Endocrine System Diseases/ |
| 3. PROMs Filter[(11)](https://www.zotero.org/google-docs/?OKIx47) | (((((((((((((((((((((((((((instrumentation or methods).sh. or Validation Studies.pt. or Comparative Study.pt. or psychometrics/ or psychometr*.ti,ab,kw. or clinimetr*.tw. or clinometr*.tw. or outcome assessment health care/ or outcome assessment.ti,ab,kw. or outcome measure*.tw. or observer variation/ or observer variation.ti,ab,kw. or Health Status Indicators/ or reproducibility of results/ or reproducib*.ti,ab,kw. or discriminant analysis/ or reliab*.ti,ab,kw. or unreliab*.ti,ab,kw. or valid*.ti,ab,kw. or coefficient.ti,ab,kw. or homogeneity.ti,ab,kw. or homogeneous.ti,ab,kw. or internal consistency.ti,ab,kw. or cronbach*.ti,ab,kw.) and alpha.ti,ab,kw.) or alphas.ti,ab,kw. or item.ti,ab,kw.) and correlation*.ti,ab,kw.) or selection*.ti,ab,kw. or reduction*.ti,ab,kw. or agreement.ti,ab,kw. or precision.ti,ab,kw. or imprecision.ti,ab,kw. or precise values.ti,ab,kw. or test-retest.ti,ab,kw. or test.ti,ab,kw.) and retest.ti,ab,kw.) or reliab*.ti,ab,kw.) and test.ti,ab,kw.) or retest.ti,ab,kw. or stability.ti,ab,kw. or interrater.ti,ab,kw. or inter-rater.ti,ab,kw. or intrarater.ti,ab,kw. or intra-rater.ti,ab,kw. or intertester.ti,ab,kw. or inter-tester.ti,ab,kw. or intratester.ti,ab,kw. or intra-tester.ti,ab,kw. or interobserver.ti,ab,kw. or inter-observer.ti,ab,kw. or intraobserver.ti,ab,kw. or intraobserver.ti,ab,kw. or intertechnician.ti,ab,kw. or inter-technician.ti,ab,kw. or intratechnician.ti,ab,kw. or intra-technician.ti,ab,kw. or interexaminer.ti,ab,kw. or inter-examiner.ti,ab,kw. or intraexaminer.ti,ab,kw. or intra-examiner.ti,ab,kw. or interassay.ti,ab,kw. or inter-assay.ti,ab,kw. or intraassay.ti,ab,kw. or intra-assay.ti,ab,kw. or interindividual.ti,ab,kw. or inter-individual.ti,ab,kw. or intraindividual.ti,ab,kw. or intra-individual.ti,ab,kw. or interparticipant.ti,ab,kw. or inter-participant.ti,ab,kw. or intraparticipant.ti,ab,kw. or intra-participant.ti,ab,kw. or kappa.ti,ab,kw. or kappas.ti,ab,kw. or kappas.ti,ab,kw. or repeatab*.ti,ab,kw. or replicab*.ti,ab,kw. or repeated.ti,ab,kw.) and measure.ti,ab,kw.) or measures.ti,ab,kw. or findings.ti,ab,kw. or result.ti,ab,kw. or results.ti,ab,kw. or test.ti,ab,kw. or tests.ti,ab,kw. or generaliza*.ti,ab,kw. or generalisa*.ti,ab,kw. or concordance.ti,ab,kw. or intraclass.ti,ab,kw.) and correlation*.ti,ab,kw.) or discriminative.ti,ab,kw. or known group.ti,ab,kw. or factor analysis.ti,ab,kw. or factor analyses.ti,ab,kw. or dimension*.ti,ab,kw. or subscale*.ti,ab,kw. or multitrait.ti,ab,kw.) and scaling.ti,ab,kw. and analysis.ti,ab,kw.) or analyses.ti,ab,kw. or item discriminant.ti,ab,kw. or interscale correlation*.ti,ab,kw. or error.ti,ab,kw. or errors.ti,ab,kw. or individual variability.ti,ab,kw. or variability.ti,ab,kw.) and analysis.ti,ab,kw.) or values.ti,ab,kw. or uncertainty.ti,ab,kw.) and measurement.ti,ab,kw.) or measuring.ti,ab,kw. or standard error of measurement.ti,ab,kw. or sensitiv*.ti,ab,kw. or responsive*.ti,ab,kw. or minimal.ti,ab,kw. or minimally.ti,ab,kw. or clinical.ti,ab,kw. or clinically.ti,ab,kw.) and important.ti,ab,kw.) or significant.ti,ab,kw. or detectable.ti,ab,kw.) and change.ti,ab,kw.) or difference.ti,ab,kw. or small*.ti,ab,kw.) and real.ti,ab,kw.) or detectable.ti,ab,kw.) and change.ti,ab,kw.) or difference.ti,ab,kw. or meaningful change.ti,ab,kw. or ceiling effect.ti,ab,kw. or floor effect.ti,ab,kw. or Item response model.ti,ab,kw. or IRT.ti,ab,kw. or Rasch.ti,ab,kw. or Differential item functioning.ti,ab,kw. or DIF.ti,ab,kw. or computer adaptive testing.ti,ab,kw. or item bank.ti,ab,kw. or cross-cultural equivalence.ti,ab,kw. |
| 4. Exclusion Criteria | NOT animals/  NOT adults/ |
